# Supplementary figures and images for: Cochlear Synaptopathy Changes Sound-Evoked Activity Without Changing Spontaneous Discharge in the Mouse Inferior Colliculus
Source: Front Syst Neurosci. 2018 Dec 3;12:59. doi: 10.3389/fnsys.2018.00059 (PMC6286982; doi:10.3389/fnsys.2018.00059)

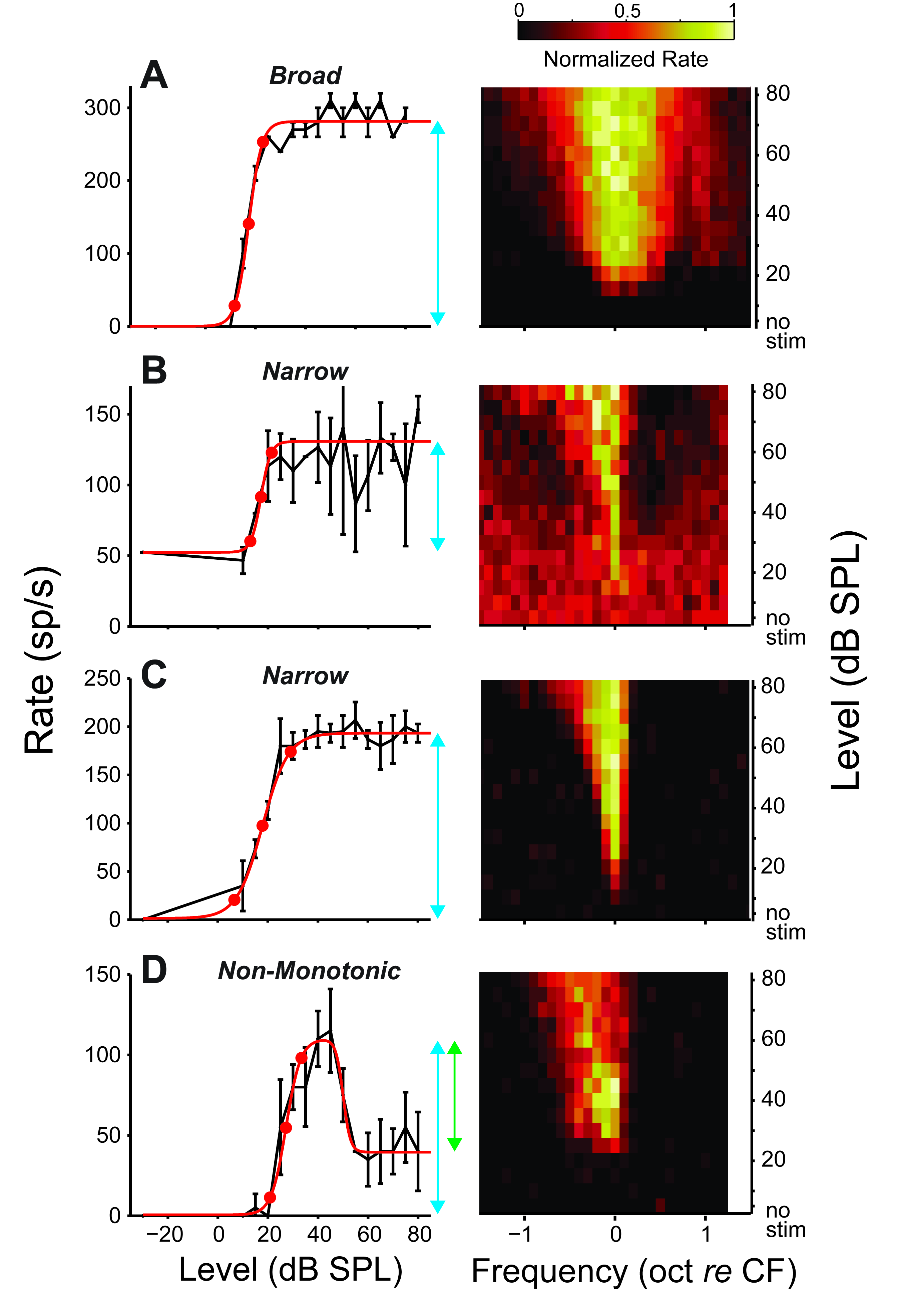

Supplement: FIGURE S1 — Exemplars of four IC unit types based on frequency response areas. (A–D) Right column shows frequency response areas, color indicates firing rate, normalized to maximum rate. Left column shows mean rate-vs.-level functions (±SEMs) to CF tones (black) with red line showing best-fit functions with 10, 50, and 90% of the excitatory driven rate range (blue arrow) indicated by red circles. Inhibitory driven rate is indicated by green arrow. [file Image_1.TIF]

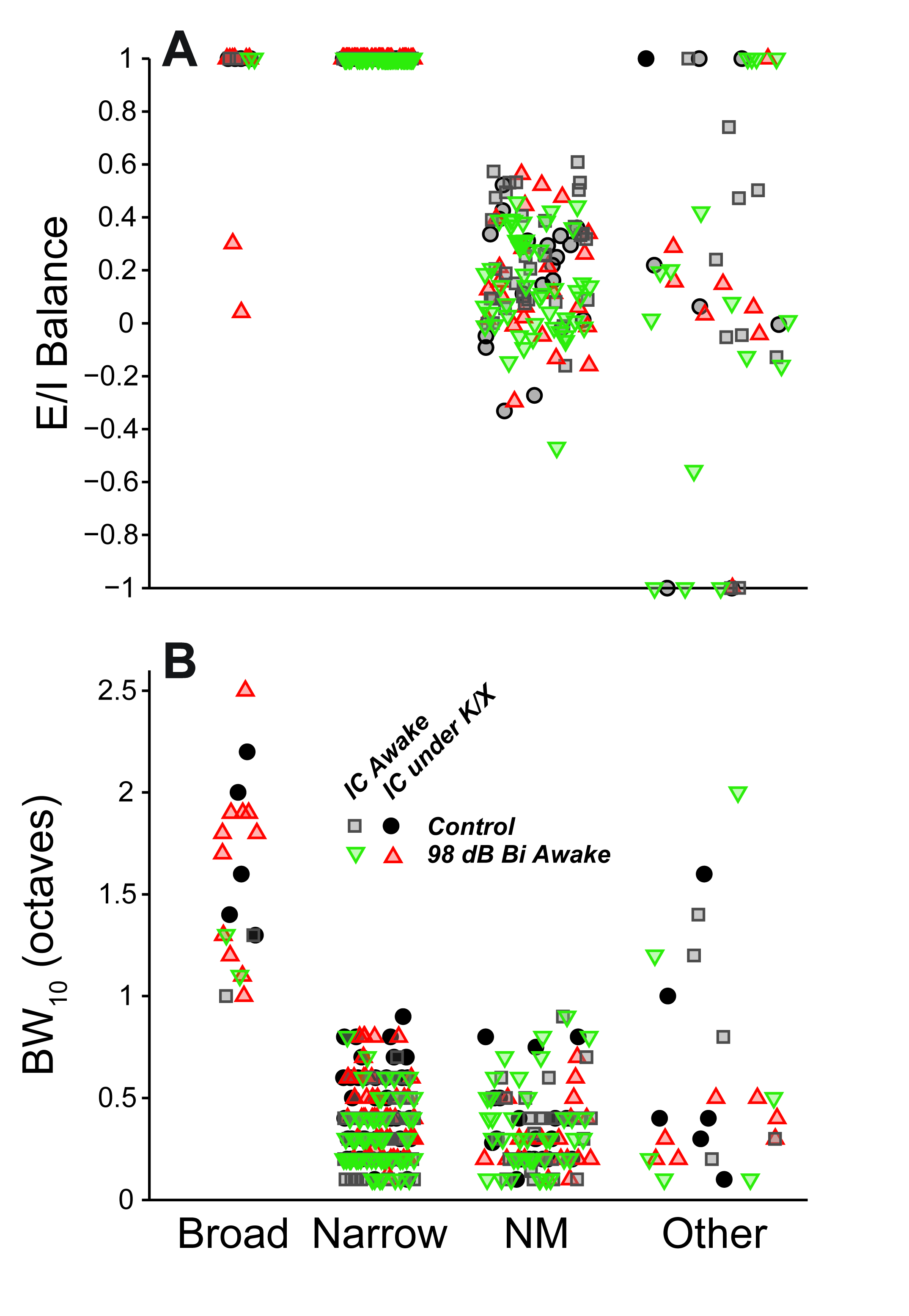

Supplement: FIGURE S2 — Metrics used for unit typing based on frequency response areas. (A) Excitatory/Inhibitory balance, the difference between excitatory and inhibitory driven rates (see Supplementary Figure S1) normalized by their sum. (B) The bandwidth of the excitatory response at 10 dB above threshold. [file Image_2.TIF]

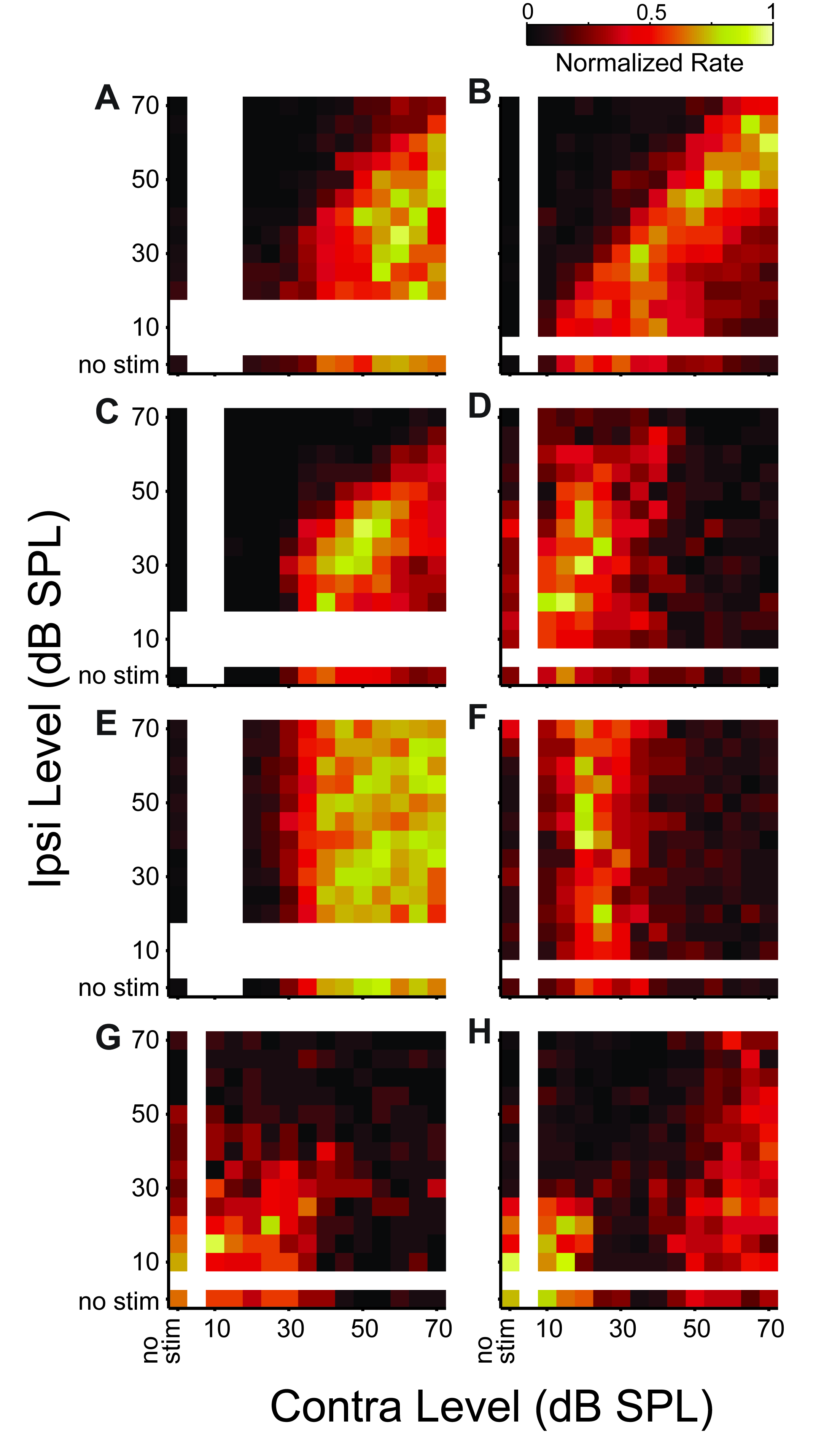

Supplement: FIGURE S3 — Unit types based on binaural-noise response areas, with normalized rate (to maximum rate) for 8 control exemplars. (A) Excitatory/Inhibitory (EI). (B) Excitatory/Inhibitory (EI), contra-non-monotonic. (C,D) Excitatory/Inhibitory with ipsilateral facilitation (EI/f), contra-non-monotonic. (E) Excitatory/No Response (EO). (F) Excitatory/No Response (EO), contra-non-monotonic. (G) Inhibitory/Inhibitory (II). (H) Inhibitory/Inhibitory (II), contra-non-monotonic. [file Image_3.TIF]

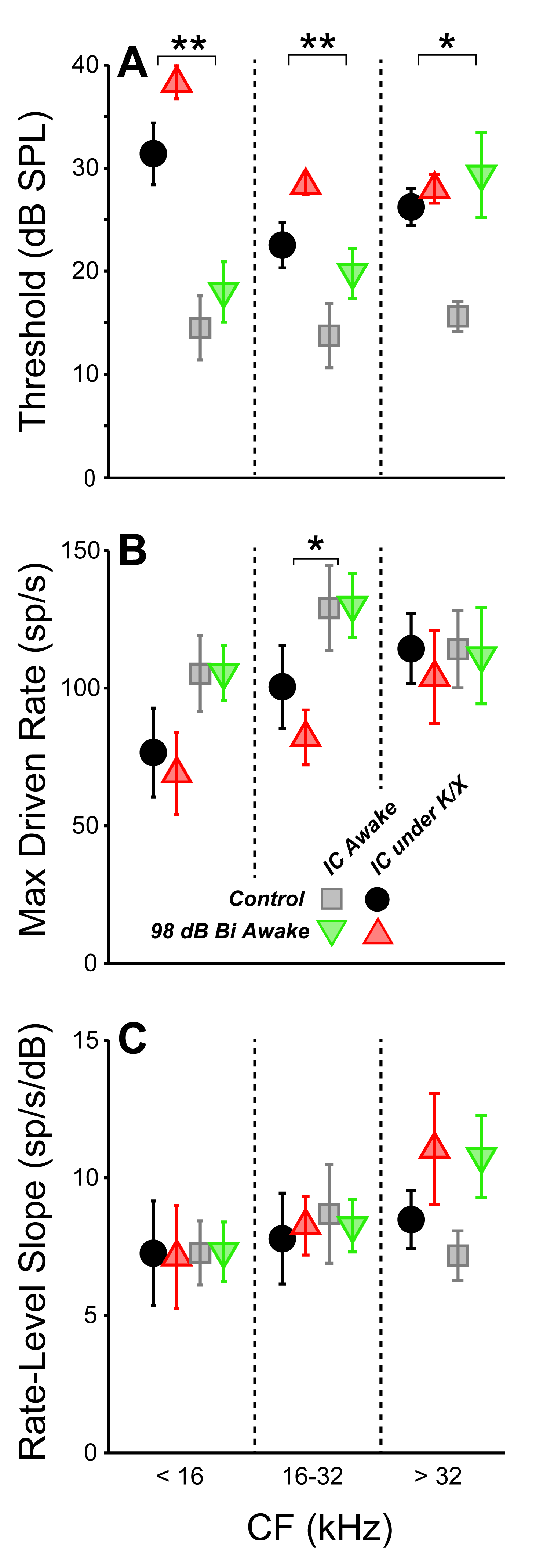

Supplement: FIGURE S4 — IC Single-unit responses to noise were unchanged following neuropathic damage. Mean threshold (A), maximum driven rate (B), and slope of the rate-level functions (C) for contralateral broadband noise, extracted from the binaural noise response maps. Black asterisks indicate significant effects of anesthesia as described in Figure 4. There were no significant effects of exposure. No effects of exposure on spontaneous or sound-evoked rates were revealed by classifying neurons by tone-, binaural-noise, or contralateral-noise response types (data not shown). [file Image_4.TIF]
